# Effective inhibition of *Clostridioides difficile* by the novel peptide CM-A

**DOI:** 10.1371/journal.pone.0257431

**Published:** 2021-09-13

**Authors:** Sirirak Arthithanyaroj, Surang Chankhamhaengdecha, Urai Chaisri, Ratchaneewan Aunpad, Amornrat Aroonnual

**Affiliations:** 1 Department of Tropical Nutrition and Food Science, Faculty of Tropical Medicine, Mahidol University, Bangkok, Thailand; 2 Department of Biology, Faculty of Science, Mahidol University, Bangkok, Thailand; 3 Department of Tropical Pathology, Faculty of Tropical Medicine, Mahidol University, Bangkok, Thailand; 4 Graduate Program in Biomedical Sciences, Faculty of Allied Health Sciences, Thammasat University, Pathum Thani, Thailand; Nanyang Technological University, SINGAPORE

## Abstract

*Clostridioides difficile* infection is the most common cause of nosocomial and antibiotic-associated diarrhea. *C*. *difficile* treatment is increasingly likely to fail, and the recurrence rate is high. Antimicrobial peptides are considered an alternative treatment for many infectious diseases, including those caused by antibiotic resistant bacteria. In the present study, we identified a CM peptide, a hybrid of cecropin A and melittin, and its derivative which possesses potent antimicrobial activity against *C*. *difficile* strain 630. CM peptide exhibited antibacterial activity with minimum inhibitory concentration of 3.906 μg/ml (2.21 μM). A modified derivative of CM, CM-A, exhibited even greater activity with a minimum inhibitory concentration of 1.953 μg/ml (1.06 μM) and a minimum bactericidal concentration of 7.8125 μg/ml (4.24 μM), which indicates that CM-A peptide is more efficient than its parent peptide. A fluorescence-activated cell sorter analysis revealed that the membrane of *C*. *difficile* 630 could be an important target for CM-A. This peptide induced high levels of cell depolarization and cell permeability on *C*. *difficile* cell membrane. Moreover, electron microscopy imaging showed that CM-A interferes with the *C*. *difficile* cell membrane. Hence, the antimicrobial peptide CM-A may represent a promising novel approach for the treatment of *C*. *difficile* infections.

## Introduction

*Clostridioides difficile* is a Gram-positive, strictly anaerobic, and spore-forming bacterium. *C*. *difficile* infection (CDI) can cause *C*. *difficile*-associated disease (CDAD) and antibiotic-associated diarrhea [1, 2]. The clinical symptoms range from mild to serious diarrhea, pseudomembranous colitis, and death [3–5]. The risk factors for *C*. *difficile* infection frequently include antibiotic usage, exposure to environmental contamination, and long-term hospital stays [6–9]. Either vancomycin or fidaxomicin is recommended by the IDSA/SHEA guidelines for the treatment of *C*. *difficile* infections in the initial episodes of adult patients [10]. However, the recurrence of infection and failure of antibiotic treatment has led researchers to investigate alternative treatments [11, 12]. In previous studies, researchers examined natural antimicrobial agents, especially antimicrobial peptides (AMPs) that can be developed into alternative treatment. AMPs are peptides comprising less than 100 amino acid residues (typically 6–100 amino acids) found in prokaryotes and eukaryotes, including insects, humans, other animals, and microorganisms [13, 14]. AMPs are associated with innate immunity and demonstrate a broad spectrum of antimicrobial activity. Most of AMPs are positively charged because of their arginine and lysine residues [15] resulting in the formation of a pore in the bacterial cell membrane, which depolarized the membrane and leads to cell death [15]. They are categorized into four classes according to their secondary structure, including *β*-sheets, *α*-helices, loops, and extended peptides [15–17]. The various structures and amino acid residues of the peptides play important roles in the inhibition of bacteria, fungi, viruses, and cancer cells. In particular, α-helical and *β*-sheet peptides comprising cysteine-rich sequences exhibit strong antibacterial activities [18]. These two groups of antimicrobial peptides may act as alternatives to traditional antibiotics for *C*. *difficile* infections, especially considering the limited number of studies involving antimicrobial peptides and *C*. *difficile*. New antimicrobial agents can be developed for more active and stable variants upon the design and chemical synthesis of analogs of natural antimicrobial peptides [19, 20]. The synthesis of hybrid peptides containing different antimicrobial properties is an interest approach to obtain novel AMPs [21, 22]. CM peptide is a hybrid α-helical peptide of cecropin A and melittin. Cecropin was isolated from pig intestine and broad-spectrum antibacterial peptides [23]. It composes of two domains as C-terminus and N-terminus. The C-terminus is more hydrophobic helix and the N-terminus is strongly basic [24]. For improvement antimicrobial activity of cecropin A, the α-helical 1–8 region was usually utilized in hybridizing with others to obtain peptides [24, 25]. Melittin is a peptide from bee venom. It showed antibacterial, anti-tumor, and anti-inflammatory activities [26]. It composes of 26 amino acid residues. Nevertheless, it has limited for its strong toxic to eukaryotic cells [27]. The synthetic hybrid peptide of cecropin A—melittin (CM peptide) can improve antimicrobial activity spectrum than that of cecropin A or melittin peptides [28]. The peptide showed a broad-spectrum bactericidal activity and exerted antibacterial effects on microorganisms such as *Pseudomonas aeruginosa*, *Acinetobacter baumannii*, and *Escherichia coli* [19, 29]. Previous studies showed that the crucial physicochemical properties of AMPs such as structure, length, net charge, sequence and hydrophobicity can improve antimicrobial activities while reducing undesirable cytotoxic effects of AMPs [30]. Thus, in this study, we modified the amino acid residues of the CM peptide to improve its inhibitory effect on *C*. *difficile*. The results of this study can aid the development of AMP-based novel or derivative treatment against *C*. *difficile* infections in the future.

## Materials and methods

### Bacterial strains and culture conditions

*Clostridioides difficile* strain 630 (kindly provided by Prof. Nigel Minton, University of Nottingham, UK*)* was cultivated in brain heart infusion (BHI, Himedia, India) medium supplemented with 0.1% (w/v) taurocholate and 0.75% (v/v) thioglycolate at 37°C for 2–3 days under anaerobic cabinet. The pre-culture was prepared by inoculating BHI broth with a single, well-isolated colony and incubated at 37°C for 16–18 hours. The pre-culture was added to the BHI broth at a concentration of 1% and grown at 37°C prior to use.

### Antimicrobial peptide synthesis

**Fig 1:**
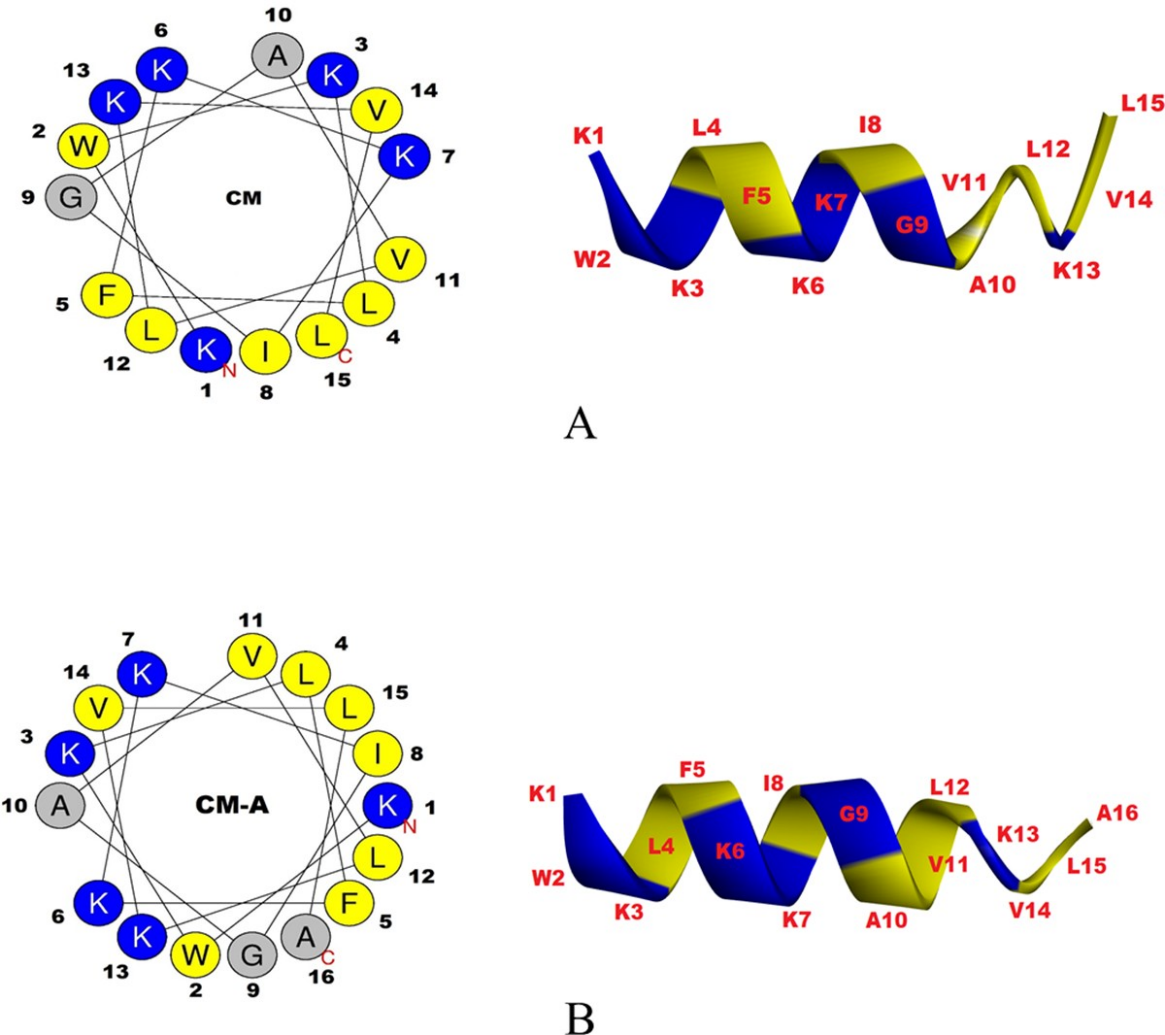
Structures of synthetic antimicrobial peptide CM and CM-A. Helical wheel projections and three-dimensional structure of CM peptide (A) and CM-A peptide (B). Hydrophobic residues are presented in yellow while positive charged residues are shown in blue. The positions of amino acid residues are presented by number.

The synthetic CM and CM-A peptides (Table 1) were synthesized via Fmoc/tBu solid phase synthesis by China Peptide (Shanghai, China). The molecular mass and purity of the peptides were verified by mass spectroscopy and reverse phase high-performance liquid chromatography (RP-HPLC), respectively. The purity of the peptides was greater than 98%. The helical wheel projection and additional physicochemical properties, including the hydrophobicity and the net charge at neutral pH, were determined using Heliquest (http://heliquest.ipmc.cnrs.fr/cgi-bin/ComputParamsV2.py) [31]. A three-dimensional structure of the peptide CM and CM-A was predicted by I-TASSER (http://zhanglab.ccmb.med.umich.edu/I-TASSER/) as shown in Fig 1. The stock solutions were freshly prepared in dimethyl sulfoxide (DMSO; Merck, USA) at a concentration of 10 mg/ml. Peptides used in the experiment were two-fold diluted and concentrations were varied ranging 0.488 to 62.5 μg/ml.

**Table 1:** Amino acid sequences and properties of synthetic CM and CM-A.

Peptide	Sequence	Net charge	Hydrophobicity (%)
CM	KWKLFKKIGAVLKVL-NH2	+5	60
CM-A	KWKLFKKIGAVLKVLA-NH2	+6	62

### Determination of the minimum inhibitory concentration (MIC) and minimum bactericidal concentration (MBC) of the peptides

The MIC was defined as the lowest concentration of a compound that inhibits the growth of tested organisms. An inoculum of *C*. *difficile* 630 (10^6^ CFU/ml) was tested with peptides at various concentrations. The MIC of the CM and CM-A peptides was determined using 96-well microtiter plates. Peptides were two-fold diluted to obtain concentrations of 62.5, 31.25, 15.625, 7.8125, 3.906, 1.953, 0.976, and 0.488 μg/ml (0.28–35.30 μM for CM peptide and 0.27–33.94 μM for CM-A peptide). *C*. *difficile* inoculum (1%) was transferred into BHI broth (300 ml) and cultivated for 6 hours at 37°C under anaerobic conditions. One hundred microliters of the culture were transferred into a 96-well plate. The CM and CM-A peptides (100 μl) with various concentrations were also added into the well. Vancomycin was used as a positive control. After 24 hours of incubation, the optical density at 600 nm was measured using a microplate reader (Tecan Sunrise^TM^, Tecan Group Ltd, Switzerland). MIC values represented the lowest concentration of CM and CM-A peptides that inhibited the growth of *C*. *difficile* 630 under the tested conditions. The MBC was defined as the lowest concentration of an antibacterial substance that kills ≥99.9% of the bacteria. After 24 hours of incubation, the mixture of *C*. *difficile* and the CM or CM-A peptide was used to inoculate onto BHI agar plates. The MBC of the peptides was investigated by observing the viability of the bacteria on the agar plate after incubation for 24 hours at 37°C under anaerobic conditions. The experiments were performed in triplicate.

### Cytotoxicity assays

The human colon adenocarcinoma cell line (Caco-2, HBT37) was used to investigate the cytotoxicity of CM-A peptide. Dulbecco’s modified Eagle’s medium (DMEM) (Hyclone, USA) containing 10% fetal bovine serum was supplemented with 1% penicillin and streptomycin (Hyclone, USA). Ten milliliters of media were added into T75 flasks for the culture of Caco-2 cells at 37°C in the presence of 5% CO_2_. Cells were grown until 70%-80% confluence prior to testing [31, 32]. A concentration of 3×10^4^ cells/well of Caco-2 cells were seeded into a 96-well plate and incubated at 37°C for 24 hours. Culture supernatants were removed. CM-A peptide and DMEM were then added into the 96-well plate at a ratio of 1:1. After 24 hours, 0.5 mg/ml of 3-(4,5-dimethylthiazol-2-yl)-2,5-diphenyltetrazolium bromide (MTT) solution (Calbiochem, St. Louis, USA) was added into each well. The mixture was incubated for 2 hours in a dark room. The reaction was stopped using one hundred microliters DMSO. DMEM with bacterial medium (tryptic soy agar) was used as negative control). The absorbance of the reaction solution was measured using microplate reader (Biofil, Elgin, USA) at a wavelength of 570 nm. The viability of Caco-2 cells was calculated by comparing the OD of Caco-2 incubated with TSB broth with that of the Caco-2 cells treated with the peptides. The experiments were performed in triplicate.

### Flow cytometry

Flow cytometry was used to investigate the effects of CM-A peptide on the permeability and membrane potential of *C*. *difficile* 630. Membrane permeability and membrane potential were tested using propidium iodide (PI) and bis-(1, 3-dibutylbarbituric acid) trimethine oxonol (DiBAC4) (Calbiochem, St. Louis, USA), respectively [3]. BHI broth was inoculated with 1% *C*. *difficile* 630 pre-culture and was then continually grown for 6 hours at 37°C under anaerobic conditions. Bacterial cells were harvested by centrifugation at 5,000 rpm for 5 minutes, and pellets were re-suspended in two milliliters of BHI broth. The CM-A peptide were added into the solution at a concentration of 4 times of the MIC (4xMIC), while the positive control was heated at 70°C for 30 minutes. PI and DiBAC4 were then added into the solution at final concentrations of 20 μg/ml and 5 μM, respectively. After incubation for 10 minutes, the mixtures were centrifuged at 4,500 rpm for 10 minutes. The pellets were collected and resuspended in four hundred milliliters of phosphate buffer saline (PBS) with a pH of 7.4. The fluorocytometric analyses were performed using flow cytometry with a BD FACSCanto Cell Analyzer (BD Biosciences, Belgium). The number of fluorescence events versus the fluorescence of the population were plotted as a histogram using FACSDiva version 6.1.1 [33, 34]. The experiments were performed in duplicate.

### Transmission electron microscopy

The membrane structure of *C*. *difficile* after treatment with the CM-A peptide was visualized via transmission electron microscopy. Following peptide treatment, *C*. *difficile* pellets were fixed with 2.5% glutaraldehyde for 1 hour and then washed three times with sucrose phosphate buffer. For postfixation, cells were re-suspended in 1% osmium tetraoxide, incubated for 1 hour, washed three times with sucrose phosphate buffer, and dehydrated with increasing concentrations of alcohol (30%, 50%, 70%, 90%, and 100%). Then, samples were embedded with resin and cut with an ultramicrotome (Leica UC7, Germany). Sections were strained with uranyl acetate and lead citrate. Ultrathin sections were visualized using a HT7700 transmission electron microscope (Hitachi Global Company, Japan) [35, 36].

### Statistical analysis

In this study, the data was analyzed by SPSS program version 22.0 was used for analysis. Results are presented as mean ± standard deviation (SD) for MIC and cytotoxicity. T-tests were used to evaluate differences in cell viability between each concentration of peptides and control. A p-value ≤ 0.05 was considered statistically significant.

## Results

### Design of a novel short peptide derivative

Amino acid sequences and properties of short peptide (CM) and its derivative (CM-A) are shown in Table 1. CM and CM-A peptides compose of 15 amino acid and 16 amino acid residues with positive charge of +5 and +6, respectively. CM-A peptide was designed by adding one alanine residue to the C-terminus (Fig 1). This alanine was added at position 16 because this position can link the hydrophobic and hydrophilic faces of the peptide. This modification improves the secondary structure, increases positive charge and increases hydrophobicity.

### Antimicrobial activity of CM and CM-A peptides against *C*. *difficile*

**Fig 2:**
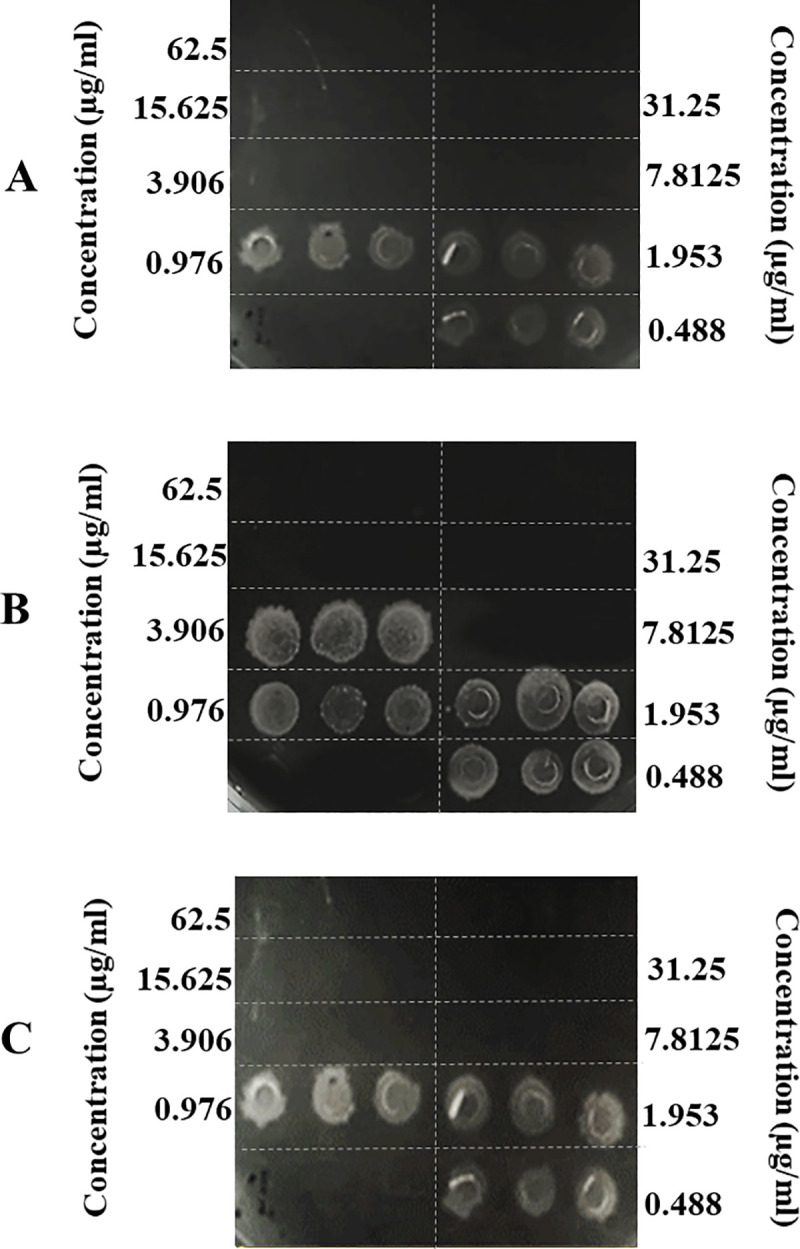
MBC of CM and CM-A peptides on *C*. *difficile* 630. *C*. *difficile* was incubated with various concentrations of CM (A) and CM-A (B) peptides. After incubation for 24 hours at 37°C under anaerobic condition, the mixture was stamped on BHI agar. The viability of the *C*. *difficile* was observed. Vancomycin (C) was used as positive control. Each condition was done in triplicate.

*C*. *difficile* was treated with various concentrations of CM and CM-A peptides to evaluate the inhibitory effects of peptides. MIC values of the tested peptides are shown in Table 2. CM peptide showed antimicrobial activity against *C*. *difficile* at MIC of 3.906 μg/ml (2.21μM). CM-A was more effective than CM with MIC of 1.953 μg/ml (1.06 μM). However, CM and CM-A peptides were less effective compared with that of vancomycin, positive control. The minimum bactericidal concentration (MBC) of CM and CM-A was 3.906 μg/ml and 7.8125 μg/ml (2.21μM and 4.24 μM), respectively (Fig 2). Based on the greater efficacy of the CM-A, this peptide was selected for further investigations into its effects on *C*. *difficile*.

**Table 2:** The MIC values of CM and CM-A peptides against *C*. *difficile* 630.

Peptides	MIC (μg/ml)	MIC (μM)
CM	3.906 ± 0.001	2.21
CM-A	1.953 ± 0.002	1.06
Vancomycin	0.488 ± 0.001	0.34

### Cytotoxicity assay

**Fig 3:**
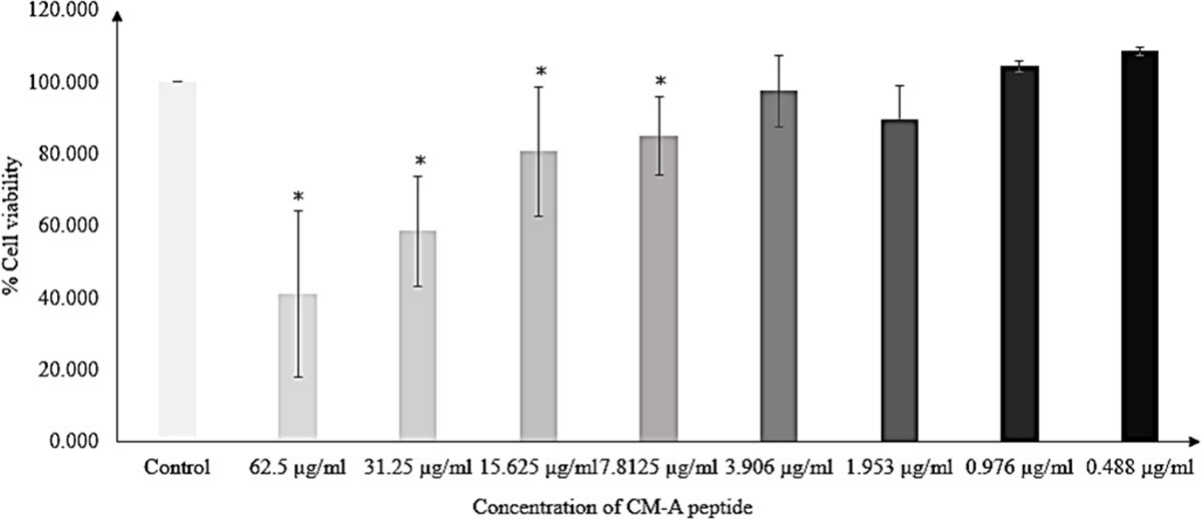
Effect of CM-A peptide on the viability of Caco-2 cells investigated by a MTT assay. Bars denoted by an asterisk (*) indicate a significant difference (p < 0.05) in comparison to the control. The experiments were performed in triplicate.

The effect of CM-A peptide on the viability of Caco-2 cells was investigated using a 3-(4,5-dimethylthiazol-2-yl)-2,5-diphenyltetrazolium bromide tetrazolium (MTT) assay. Viability of Caco-2 cells reduced when the cells were treated with higher concentrations of CM-A. At a MIC of 1.953 μg/ml (1.06 μM), viability of Caco-2 cell reduced by 11%. Statistical analyses (t-test) showed no significant differences compared with that of the control as shown in Fig 3. However, the increasing of CM-A concentration to 62.5 μg/ml (33.94 μM) can reduce viability of Caco-2 cells to 40%. This finding indicates that the use of this peptide at its MIC concentration was not toxic to the Caco-2 cells.

### Determination of the membrane potential and permeability of *C*. *difficile* by treating with CM-A peptide

**Fig 4:**
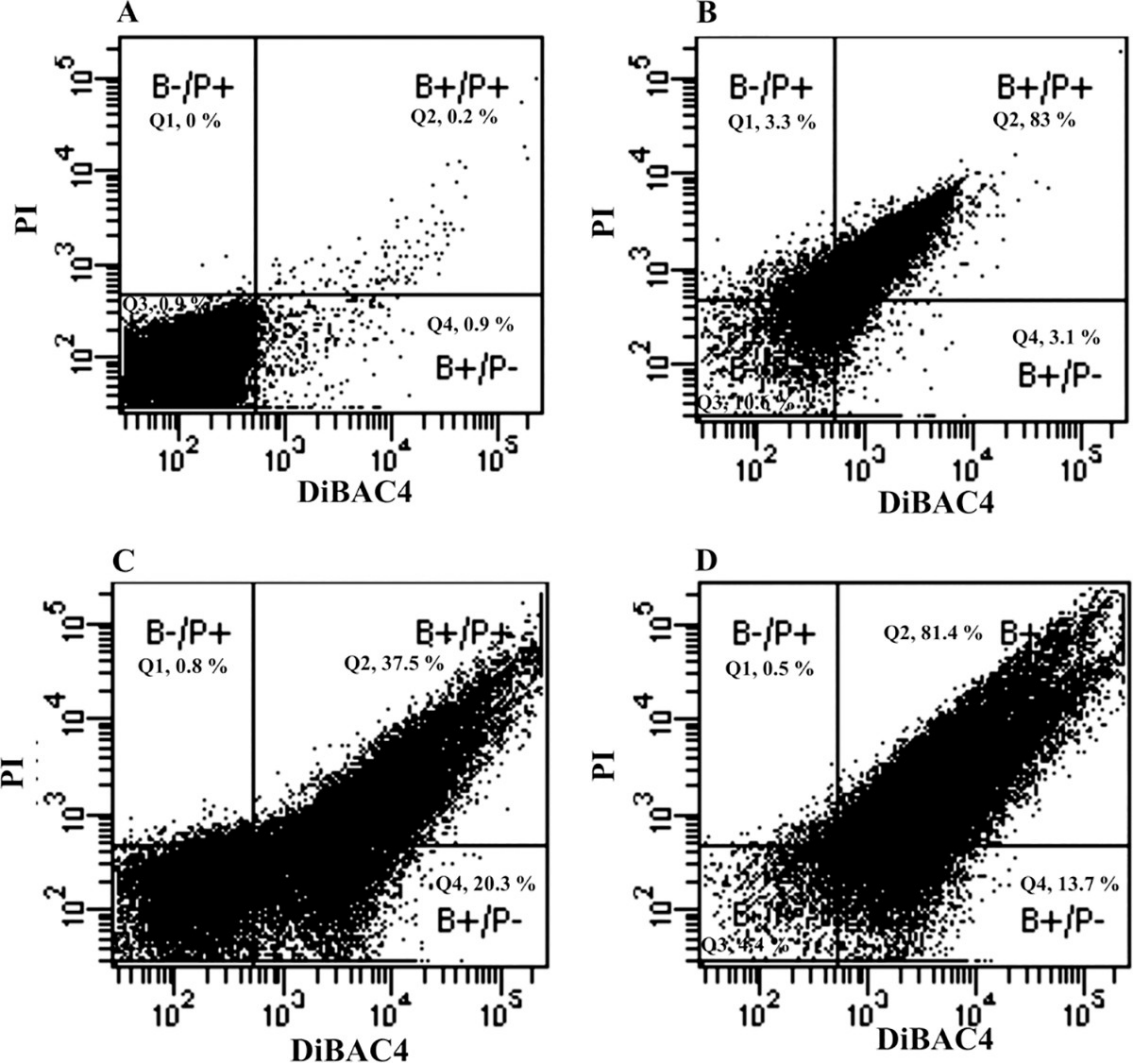
Fluorescence dot plot of *C*. *difficile* stained with PI and DiBAC4. (A) Untreated *C*. *difficile*. (B) *C*. *difficile* heated at 70°C. (C) *C*. *difficile* treated with 1X MIC of CM-A. (D) *C*. *difficile* treated with 4X MIC of CM-A.

Two fluorescent dyes were used to evaluate the bactericidal activity of the peptide, CM-A, against *C*. *difficile*. Dead cells can be visualized with propidium iodide (PI), which acts as a nucleic acid intercalating agent. DiBAC4, another dye, can enter into cells and detect membrane depolarization caused by injury, stress, or cell death. The fluorescence dot plots of *C*. *difficile* stained with PI and DiBAC4 are shown in Fig 4. The dot plot of untreated cells (Fig 4A) showed that most of the untreated *C*. *difficile* cells were not stained with both PI and DiBAC4 (Q1) indicating no cell damage. When *C*. *difficile* 630 was heated (Fig 4B), more florescence dots were observed in the B+/P+ panel than in those of untreated cells, which indicates that the bacterial cells were damaged and were stained with both fluorescent dyes. Treatment of *C*. *difficile* with the CM-A peptide with the concentration at MIC (Fig 4C) and at 4x MIC (Fig 4D) exhibited an increase of inhibitory effect of the peptide to *C*. *difficile* cells, as shown by the increased number of fluorescent dots in panel Q2. The percentage of damaged *C*. *difficile* cells increased to 37.5% and 81.4% when treated with CM-A at the MIC and 4x MIC, respectively. These results indicate that CM-A can damage the *C*. *difficile* cell membrane, which causes membrane depolarization and bacterial cell lysis.

### Investigating the effect of CM-A peptide on the cell membrane of *C*. *difficile*

**Fig 5:**
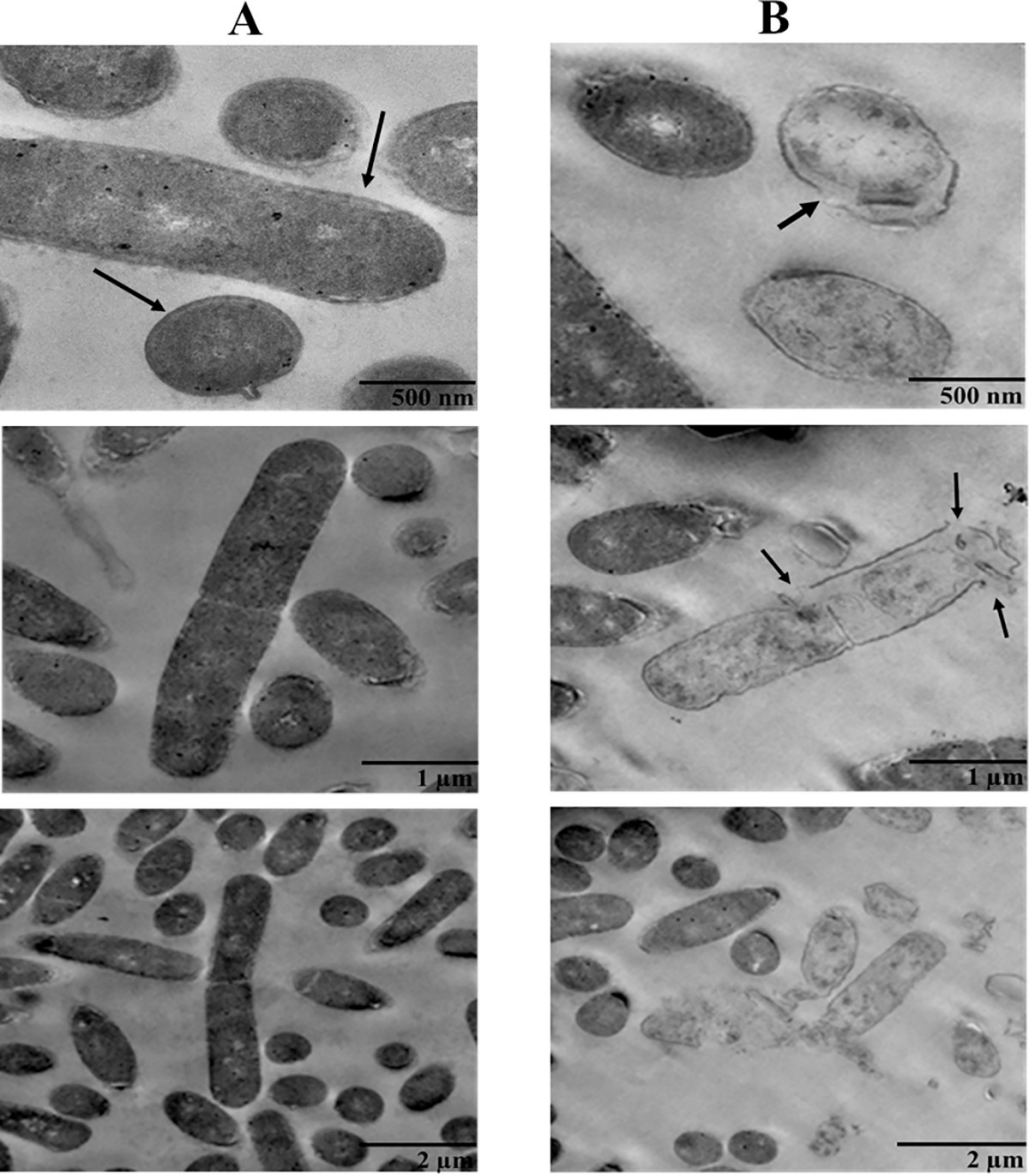
Membrane structure of *C*. *difficile* via transmission electron microscopy. (A) Untreated *C*. *difficile*. (B) *C*. *difficile* treated with CM-A peptide at 4xMIC. Arrows indicate the leakage on the cell membrane of *C*. *difficile*.

To investigate the inhibitory effects of the CM-A peptide on the *C*. *difficile* cell membrane, morphological structure of the *C*. *difficile* cell membrane were observed under a transmission electron microscopy (TEM). Fig 5A showed the structure of untreated *C*. *difficile* cells with no morphological damage. The double membrane was observed in untreated *C*. *difficile* cells. Treatment with the CM-A peptide at 4xMIC for 30 minutes can lyse the cell membrane and cause leakage of the cytoplasm in *C*. *difficile*. The degenerated membrane was observed in treated cells (Fig 5B), indicating that the CM-A peptide can inhibit *C*. *difficile* by altering the bacterial cell membrane.

## Discussion

*Clostridioides difficile* infection (CDI) is the most common pathogen implicated in nosocomial and antibiotic-associated diarrheal disease [1, 2]. The use of antibiotics for the treatment of CDI infection can lead to treatment failures and the recurrence of infection [11, 12]. Antimicrobial peptides (AMPs) are of interest as an alternative treatment for bacterial infections, especially in drug-resistant bacteria. In this study, hybrid peptides between cecropin A and melittin (CM peptide) and a CM derivative peptide, CM-A, were synthesized and tested for their inhibitory effect against *C*. *difficile* strain 630. The CM and CM-A peptides showed antimicrobial activity with MIC of 3.906 μg/ml (2.21 μM) and 1.953 μg/ml (1.06 μM), respectively. The MIC of our peptides was comparable to that of several antimicrobial peptides have been reported to inhibit the growth of *C*. *difficile* [17, 37]. Nisin, a 34-amino-acid AMP, produced by *Lactococcus lactis*, showed a MIC range from 0.8 to 6.2 μg/ml, while thuricin CD, produced by *Bacillus thuringiensis* isolated from human intestinal flora, exhibited a MIC of 0.125–0.5 μM [38]. A new technology on hybrid peptide is one of the most effective techniques used to design new AMPs with better properties than their parent peptides. Jindal HM and colleagues have reported that hybrid peptides derived from ranalexin and indolicidin inhibited *Streptococcus pneumonia*. Four of these hybrid peptides (RN7-IN10, RN7-IN9, RN7-IN8, and RN7-IN6) exhibited the strongest antipneumococcal activity [39]. In our study, the peptide CM-A exhibited a more potent antibacterial activity than that of the parental peptide. This difference may be the result of an increased hydrophobicity of the peptide via the addition of alanine at position 16. It could improve secondary structure by linking hydrophilic and hydrophobic regions in the structure. This result was in agreement with the study of Klubthawee N that truncation and amino acid substitution of P7, derivatives peptide from α-helical cathelicidin (P0), showed significantly improved antimicrobial activity. This result may be from higher hydrophobicity and amphipathicity of peptide [40]. In this study, the MIC value from CM-A peptide was lower than that of other studies such as the cathelicidin-derived peptide, SMAP-29 deduced from sheep myeloid mRNA showed antimicrobial activity against *C*. *difficile* with MIC and MBC at 2.4 mg/L and 4.8 mg/L, respectively [41]. SMAP-29 is α-helical conformation which is similar toward CM-A peptide. The 28 residue peptide of SMAP-29 has a net charge +10 [42] while 16 amino acid residue of CM-A has a net charge +6. It could be indicated that CM-A peptide had a more antimicrobial activity toward *C*. *difficile* than SMAP-29. Moreover, coprisin, a peptide from *Copris tripartitus*, showed inhibitory effect against *C*. *difficile* ATCC 43255 with MIC of 1.5 μg/ml [17] which was lower than that of CM-A peptide. The α-helical structure of Coprisin is like with CM-A but coprisin is adding β-sheet into the structure [42, 43]. Therefore, the various structures and amino acid residues of the peptides may play an important role in the inhibition of bacteria [18]. Although, in this study, the MIC against *C*. *difficile* of CM and CM-A peptides was not compared with that of the parent peptides, cecropin A and melittin. Several cecropin A-melittin hybrid peptides were designed and tested for their inhibitory activity against Gram-positive and Gram-negative bacteria. The result showed that the hybrid peptides CA(1–7)M(2–9), similar sequence with CM peptide, possessed higher antimicrobial activity against *Bacillus subtilis*, *Bacillus megaterium*, *Staphylococcus aureus* and *Streptococcus pyogenes* than that of the parent peptides [44, 45]. The interaction of CM-A peptide with the membrane of *C*. *difficile* was studied by flow cytometry and transmission electron microscopy. The CM-A peptide is an antimicrobial agent that targets the cell membrane of *C*. *difficile*. We found that a higher concentration of the CM-A peptide increased deterioration of the *C*. *difficile* cell membrane investigated by flow cytometry and transmission electron microscopy. According to the findings from Nuding S et al., they revealed that the concentration of synthetic peptide, HBD3, tested with *C*. *difficile*, required 3X and 40X of MIC in flow cytometry assay and transmission electron microscopy, respectively [18, 46]. At concentration 4x MIC of the CM-A peptide resulted the strong effect that exhibited more damage on the *C*. *difficile* cell membrane and its permeability leading to high number of bacterial cells was stained with dual PI/DiBAC4 dyes. These results were in accordance with the study of Kang JK and Furci L that *C*. *difficile* treated with synthetic coprisin peptide and human-α defensin 5 (HD5) peptide can cause bacterial fragmentation and depolarization of the cell membrane [17, 47]. Cell membrane structures of *C*. *difficile* were observed using TEM after incubation with 4xMIC of CM-A peptide. The membrane perturbation and disruption of the *C*. *difficile* cell membrane was similar to the damage caused by nisin [38, 48]. In addition, the α-helical hybrid peptide CA-FO (charge of +13), which comprised cecropin A (charge of +5) (CA (1–8)) and the most potent region of fowlicidin-2 (charge of +9)(FO (1–15)), exhibited the greatest antibacterial activity against both Gram-positive and Gram-negative bacteria. Furthermore, CA-FO can also damage the membrane integrity and increase membrane permeability [49].

## Conclusions

The derivative CM-A peptide was more effective against *C*. *difficile* than that of the parental peptide, CM, resulting from the modification which linked hydrophilic and hydrophobic regions, improved secondary structure, and altered hydrophobic and positive charge. Thus, the derivative peptide CM-A could represent a promising novel approach for the treatment of *C*. *difficile* infections.
